# Supplementary material for: The essential clathrin adapter protein complex-2 is tumor suppressive specifically in vivo
Source: Nat Commun. 2025 Mar 6;16:2254. doi: 10.1038/s41467-025-57521-2 (PMC11885535; doi:10.1038/s41467-025-57521-2)
Supplement: Supplementary file 1 — Supplementary Information [file 41467_2025_57521_MOESM1_ESM.docx]

Supplementary Information:

The essential clathrin adaptor protein complex-2 is tumor suppressive specifically in vivo

Seth P. Zimmerman, Lili B. DeGraw, and Christopher M. Counter


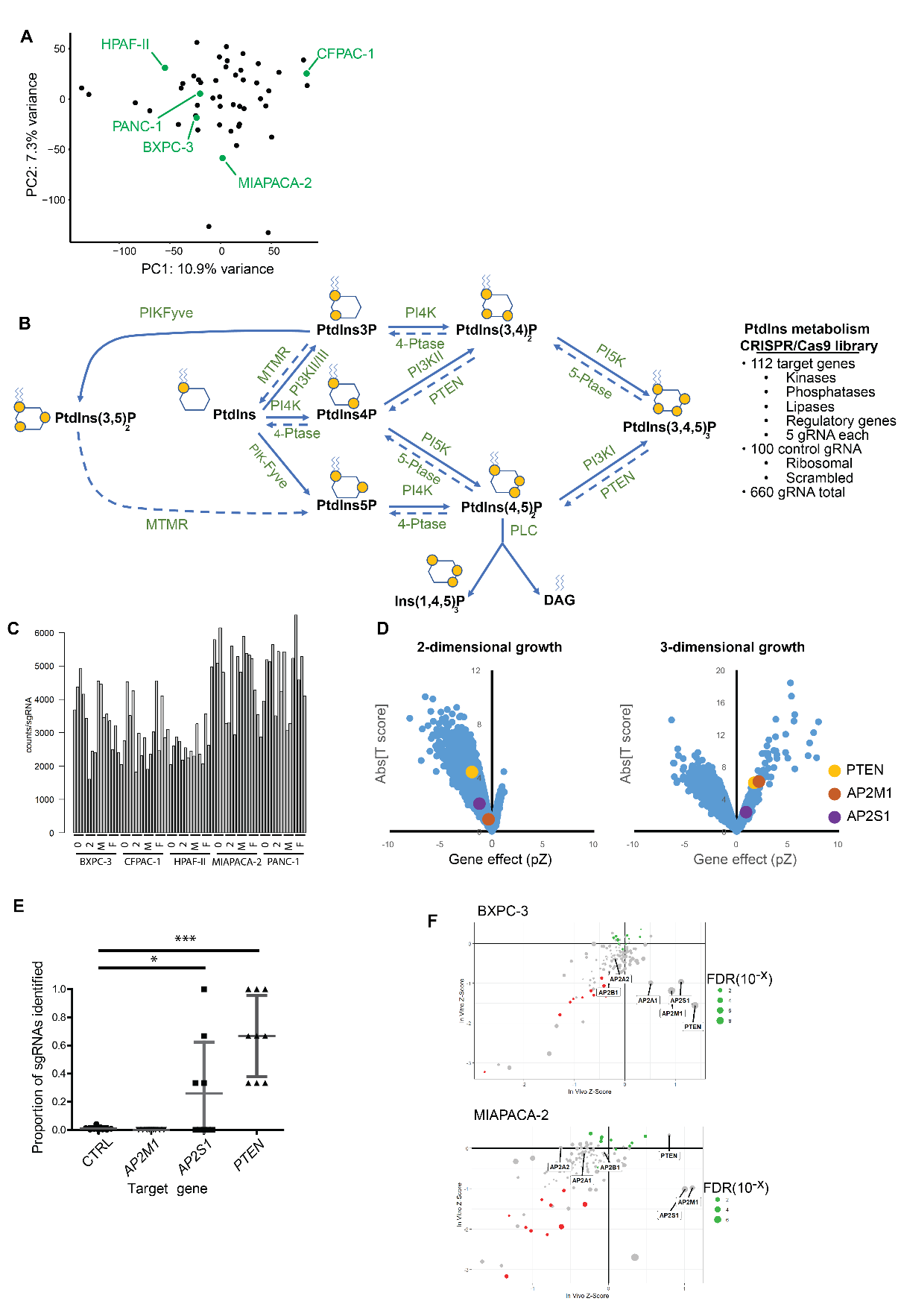


## Figure S1. PtdIns metabolism library design, quality control, and validation studies

**A**) Principal component analysis of all PDAC cell line transcriptomes contained in the DepMap^39,40^ database plotted as principal component 1 (PC1) and principal component 2 (PC2). Green highlighted cell line names were used in the study. **B**) Diagram of the PtdIns metabolism pathway adapted from^13^. Green labels are enzyme classes targeted in the library. In addition, regulatory genes were targeted. **C**) Mean *sgRNA* sequence counts per *sgRNA* in library for each screen sample plotted as Initial culture (0), two weeks of culture (2), male mouse host (M) female mouse host (F). **D**) Data mined from a whole-genome screen in H23 lung cancer cells grown in 2 and 3-dimensional culture^30^. Volcano plots represent gene loss effect (pZ = phenotype Z-score) plotted against the absolute T-score on cell growth. pZ is defined as the Z-score of fold change values centered on the negative control values. Positive pZ values indicate enhanced growth. Absolute T-score (Abs[T-score]) is defined as the absolute value of a modified T-score^30^. **E**) Data mined from a whole-genome screen in murine lung cancer model xenografts^7^. Scatter plot superimposed with mean +/- standard deviation of the proportion of gene target sgRNAs recovered from late-stage tumor growth samples. Each symbol represents one tumor (*n* = 9 tumors). The presence of *sgRNA* for a particular target indicates that the *sgRNA*-induced positive selection for the *sgRNA* and likely indicates a tumor suppressive effect for the gene. **F**) Scatter-plot of replicated in vitro (culture) and in vivo (xenograft) Z-scores representing loss-of-function gene effects from CRISPR screen. False Discovery Rates (FDRs), represented by circle size, refer to comparison of normalized in vivo to in vitro Z-Scores across tested cell lines. For each cell line, in vivo sample *n* = 3 per tumor condition, in vitro sample *n* = 3 per cell condition. **p*<0.05, ***p*< 0.01, and ****p*<0.001. Source data are provided as a Source Data file.


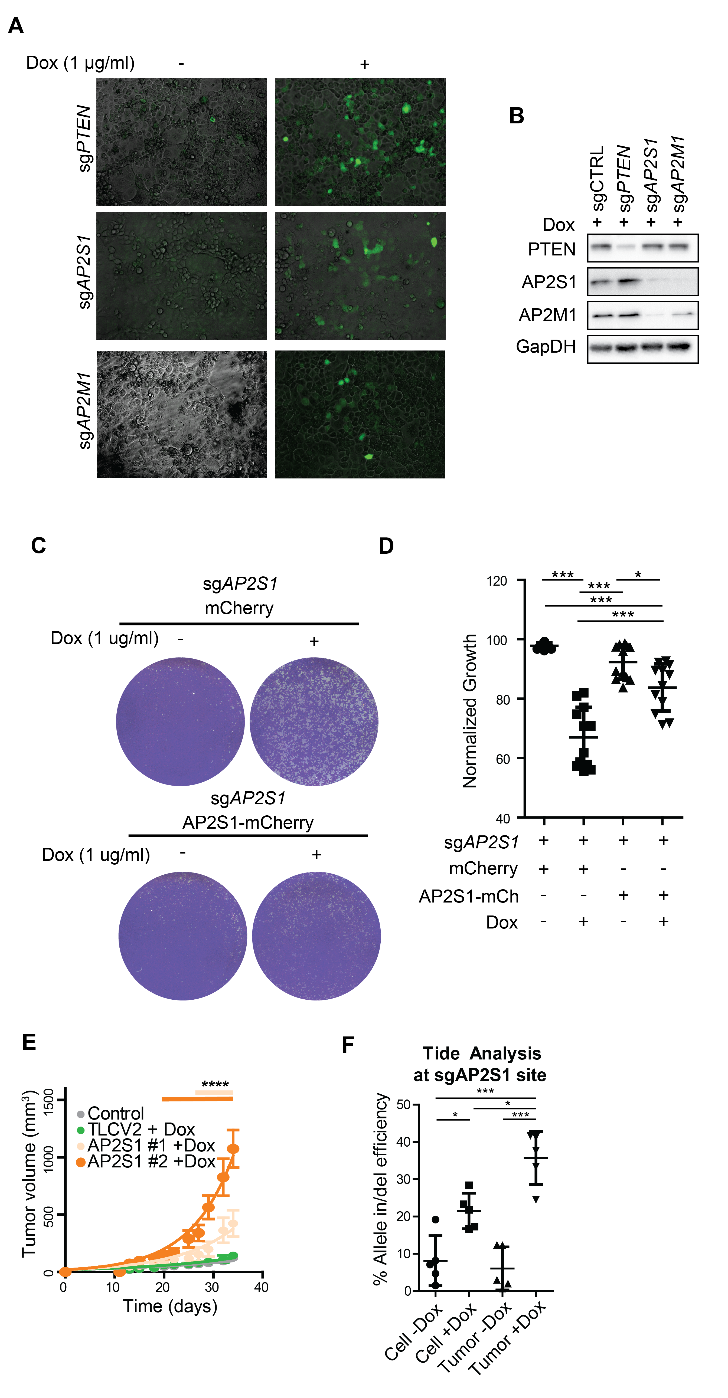


## Figure S2. BXPC-3 cell and tumor growth upon AP2S1 genetic loss.

**A**) Representative merged fluorescent and phase contrast micrographs depicting GFP expression with doxycycline (Dox) induction for indicated *sgRNA*s. **B**) Representative immunoblot analysis of the indicated proteins from BXPC-3 cells with doxycycline (Dox)-induced expression of Cas9 and the indicated *sgRNA*. (representative of n = 3 independent experiments). **C**) BXPC-3 cells visualized by crystal violet staining after 7 days of growth with doxycycline-induced *sgAP2S1* and expression of mCherry alone or *AP2S1*-mCherry. **D**) Scatter plot superimposed on mean and standard deviation of relative growth quantification from B (*n* = 12 wells total over 4 biological replicates;1-way ANOVA). **E**) Plots of tumor volume versus time in days for tumors derived from BXPC-3 cells transduced with the indicated *sgRNA*s and induced with doxycycline compared to all uninduced control tumors. Mean +/- standard error, *n* = 5 mice per condition; 2-way ANOVA. **F**) Scatter plot superimposed on mean and standard deviation of tide analysis from BXPC-3 cells and xenografts comparing insertion/deletion (in/del) efficiency at the targeted *AP2S1* genomic site with and without doxycycline induction of Cas9 (*n* = 5 cell or tumor replicates per condition; one-way ANOVA). **p*<0.05, ***p*< 0.01, ****p*<0.001, and *****p*<0.0001. Source data are provided as a Source Data file.


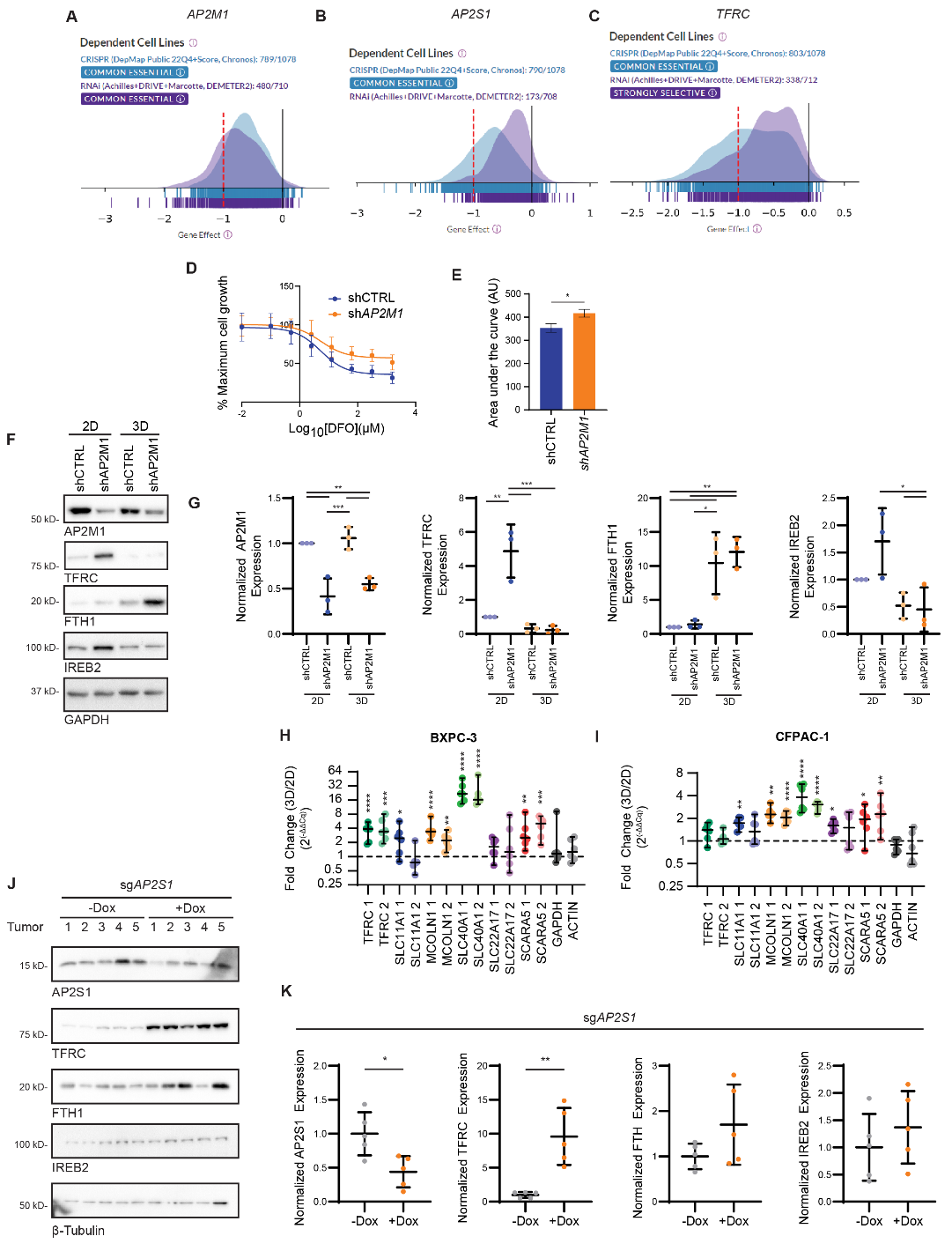


## Figure S3. Iron transport is altered by AP2 loss and cell growth environment.

**A-C**) Chronos Gene Effect^39,40^ distribution and classification across DepMap cell lines for CRISPR/Cas9 and RNAi targeting of **A**) *AP2M1*, **B**) *AP2S1*, and **C**) *TFRC*. Figure adapted from DepMap.org. **D**) DFO dose response curves for BXPC-3 cells expressing the indicated *shRNA*s. Mean +/- standard deviation is plotted. **E**) Bar plot of mean ± SD area under the curve analysis comparing the dose response curves presented in G. (*n* = 15 curves total over 5 independent experiments; two-tailed *t*-test) **F**) Representative immunoblot analysis of BXPC-3 cells grown as 2D or 3D cultures and transduced with *shCTRL* or *shAP2M1* (representative of *n* = 3 independent experiments)*.* **G**) Scatter plot superimposed on mean ± SD of immunoblots represented in F. (*n =* 3 independent experiments; one-way ANOVA). **H**) BXPC-3 and **I**) CFPAC-1 cells grown in 2D and 3D culture conditions. Each target transcript is assessed by two distinct primer pairs and GAPDH and actin serve as loading controls. (*n* = 6 technical replicates total over 2 independent experiments; t-test comparing ΔCq values). **J**) Immunoblot analysis of the indicated proteins from 10 tumors expressing *sgAP2S1* and treated or not with doxycycline (Dox) before implantation to induce Cas9. **K**) Scatter plot superimposed on mean and standard deviation of immunoblots represented in F. (*n =* 5 tumors per condition*;* two-sided *t*-test). **p*<0.05, ***p*< 0.01, ****p*<0.001, and *****p*<0.0001. Source data are provided as a Source Data file.


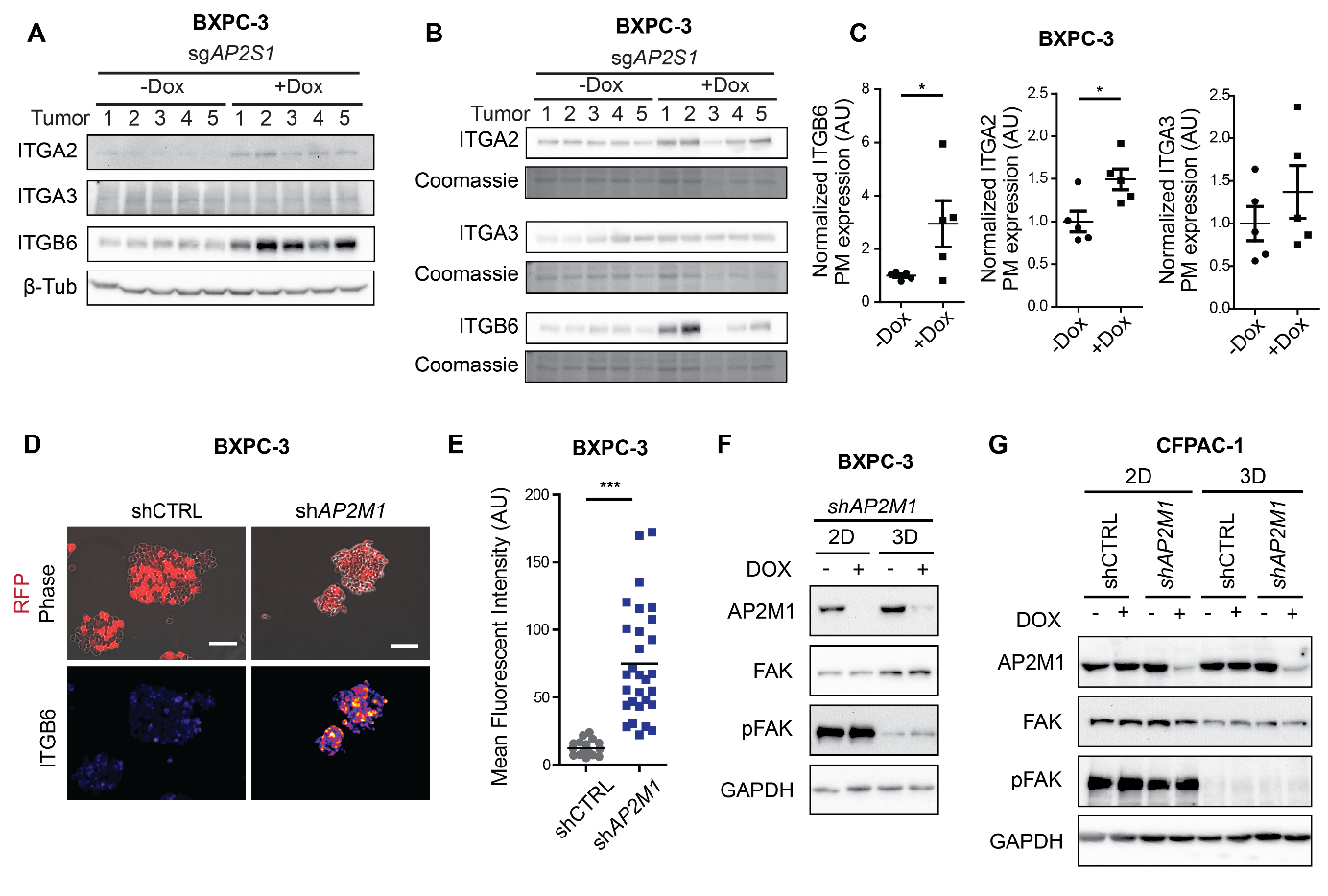


## Figure S4. *AP2S1* loss leads to increased cell membrane integrins and PI3K signaling in tumors.

**A**) Immunoblot analysis of the indicated proteins from whole-cell BXPC-3 tumor lysates. Cells were transduced with indicated *sgRNA*s and doxycycline-inducible Cas9. β-Tubulin stain serves as a loading control **B**) Immunoblot analysis of the indicated proteins from 10 tumors expressing *sgAP2S1* and treated or not with doxycycline (Dox) before implantation to induce Cas9. Protein lysates were enriched for plasma membrane proteins. Coomassie stain serves as a loading control. **C**) Scatter plot superimposed on mean ± SD of the indicated plasma membrane integrin normalized to Coomassie stain from A (*n* = 5 tumors per condition; t-test). **D**) Representative fluorescence and phase micrographs of fixed but not permeabilized BXPC-3 spheroids immuno-stained with ITGB6 antibody. RFP indicates expression of the indicated *shRNA*. (Bar = 100 µm). **E**) Scatter plot superimposed on mean ± SD of ITGB6 staining quantification from D (*shCTRL* *n* = 21 images total, *shAP2M1* *n* = 29 images total over 2 biological replicates; two-sided *t*-test). **F, G**) Representative immunoblot analysis of the indicated proteins from **F**) BXPC-3 and **G**) CFPAC cells cultured in 2D or 3D and expressing the indicated *shRNA* induced by Doxycycline. GAPDH serves as a loading control (representative of *n* = 2 independent experiments). * *p*< 0.05 and ****p*< 0.001. Source data are provided as a Source Data file.


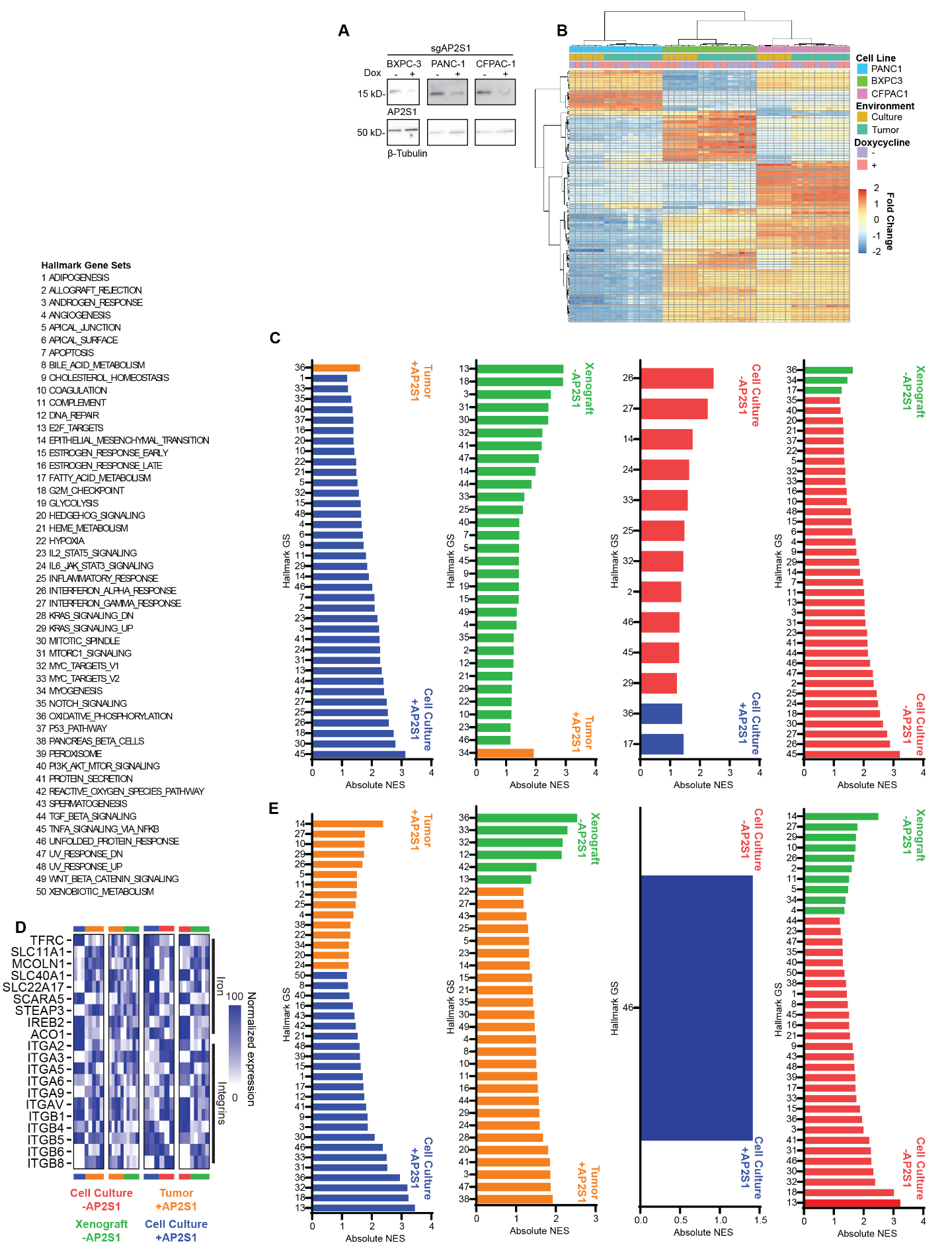


## Figure S5. Transcriptomics of PDAC models.

**A**) Immunoblot analysis of *AP2S1* in BXPC-3, PANC-1, and CFPAC-1 cells with doxycycline (Dox) induced expression of Cas9 with indicated *sgAP2S1*. β-Tubulin serves as a loading control (*n* = 1 independent experiment). **B**) A heatmap of normalized fold-change for the top 100 differentially expressed genes across each sample. X and Y axis are clustered by Ward’s Hierarchical method. Derived from RNA sequencing of the indicated cell lines, appropriate environments and with or without doxycycline. **C**) Bar graphs of the absolute normalized enrichment scores (NES) comparing gene sets enriched in BXPC-3 cells grown in culture or as tumors with or without AP2S1. (hallmark gene set indicated by number and include all gene sets with an FDR < 0.25; *n* = 5 for each tumor condition and *n* = 3 for each cell condition) **D**) Transcriptional changes in PANC-1 cells as growth environment and *AP2S1* status are changed. Heat maps represent normalized expression of manually curated genes of interest from indicated pathways (*n* = 5 for each tumor condition and *n* = 3 for each cell condition). **E**) Bar graphs of the absolute normalized enrichment scores (NES) comparing gene sets enriched in PANC-1 cells grown in culture or as tumors with or without AP2S1. (hallmark gene sets with an FDR < 0.25; *n* = 5 tumor per condition and *n*=3 for each cell condition). Source data are provided as a Source Data file.
