# Supplementary material for: The essential clathrin adapter protein complex-2 is tumor suppressive specifically in vivo
Source: Nat Commun. 2025 Mar 6;16:2254. doi: 10.1038/s41467-025-57521-2 (PMC11885535; doi:10.1038/s41467-025-57521-2)
Supplement: Supplementary file 2 — Description of Additional Supplementary Files [file 41467_2025_57521_MOESM2_ESM.pdf]

Description of Additional Supplementary Files

Supplementary Data 1: sgRNA library sequences

Supplementary Data 2: CRISPR library barode sequences

Supplementary Data 3: Normalized CRISPR screen sgRNA counts

Supplementary Data 4: Normalized Plasma Membrane Peptide Counts

Supplementary Data 5: Normalized RNA sequencing counts

Supplementary Data 6: RT-qPCR primer sequences
