# Supplementary material for: The essential clathrin adapter protein complex-2 is tumor suppressive specifically in vivo
Source: Nat Commun. 2025 Mar 6;16:2254. doi: 10.1038/s41467-025-57521-2 (PMC11885535; doi:10.1038/s41467-025-57521-2)
Supplement: Supplementary file 9 — Reporting Summary [file 41467_2025_57521_MOESM9_ESM.pdf]

nature portfolio

Corresponding author(s): Chris Counter

Last updated by author(s): Aug 9, 2024

Reporting Summary

Nature Portfolio wishes to improve the reproducibility of the work that we publish. This form provides structure for consistency and transparency in reporting. For further information on Nature Portfolio policies, see our Editorial Policies and the Editorial Policy Checklist.

Statistics

For all statistical analyses, confirm that the following items are present in the figure legend, table legend, main text, or Methods section.

n/a	Confirmed	
☐	☒	The exact sample size (*n*) for each experimental group/condition, given as a discrete number and unit of measurement
☐	☒	A statement on whether measurements were taken from distinct samples or whether the same sample was measured repeatedly
☐	☒	The statistical test(s) used AND whether they are one- or two-sided<br>*Only common tests should be described solely by name; describe more complex techniques in the Methods section.*
☒	☐	A description of all covariates tested
☒	☐	A description of any assumptions or corrections, such as tests of normality and adjustment for multiple comparisons
☐	☒	A full description of the statistical parameters including central tendency (e.g. means) or other basic estimates (e.g. regression coefficient) AND variation (e.g. standard deviation) or associated estimates of uncertainty (e.g. confidence intervals)
☐	☒	For null hypothesis testing, the test statistic (e.g. *F*, *t*, *r*) with confidence intervals, effect sizes, degrees of freedom and *P* value noted<br>*Give P values as exact values whenever suitable.*
☒	☐	For Bayesian analysis, information on the choice of priors and Markov chain Monte Carlo settings
☐	☒	For hierarchical and complex designs, identification of the appropriate level for tests and full reporting of outcomes
☐	☒	Estimates of effect sizes (e.g. Cohen's *d*, Pearson's *r*), indicating how they were calculated

Our web collection on statistics for biologists contains articles on many of the points above.

Software and code

Policy information about availability of computer code

Data collection	BioRad Image Lab 6.1 was used to capture all images of immunoblots
Data analysis	Software and Algorithms<br>Prism 9.5.1 Graphpad www.graphpad.com/<br><br>RStudio 2023.3.0 build 386 Posit Software posit.co/products/open-source/rstudio/<br><br>R v4.2.1 R Project www.r-project.org/<br><br>ImageJ 1.53t Schneider et al. imagej.net/downloads<br><br>GSEA 4.3.2 Subramanian et al.<br>https://www.gsea-msigdb.org/gsea/index.jsp<br>Tracking of Indels By Decomposition 3.3.0 Brinkman et al, Nucl. Acids Res. (2014)<br>shinyapps.datacurators.nl/tide/<br><br>g:Profiler version e109_eg56_p17_1d3191d Raudvere et al. biit.cs.ut.ee/gprofiler/gost<br><br>ggplot2 3.4.2 tidyverse ggplot2.tidyverse.org/<br><br>Pheatmap 1.0.12 CRAN - Package pheatmap (r-project.org)

DESeq2 1.40.1 Love et al. Bioconductor - DESeq2

dplyr 1.1.2 tidyverse dplyr.tidyverse.org/

Fgsea 1.26.0 Korotkevich et al. Bioconductor - fgsea

Survival 3.5-5 Therneau T. et al. cran.r-project.org/web/packages/survival/

Survminer 0.4.9 cran.r-project.org/web/packages/survminer/index.html

Fastx_barcode_splitter 0.0.13 hannonlab.cshl.edu/fastx_toolkit/download.html

Bowtie 1.0.0 bowtie-bio.sourceforge.net/index.shtml

Trim Galore 0.6.5 www.bioinformatics.babraham.ac.uk/projects/trim_galore/

Star 2.7 github.com/alexdobin/STAR

HTSeq 2.0.3 Putri et al. HTSeq · GitHub

Proteome Discoverer 2.5 Thermo Fisher www.thermofisher.com/us/en/home/industrial/mass-spectrometry/liquid-chromatography-mass-spectrometry-lc-ms/lc-ms-software/multi-omics-data-analysis/proteome-discoverer-software.html

Sequest UWPR proteomicsresource.washington.edu/protocols06/sequest.php

For manuscripts utilizing custom algorithms or software that are central to the research but not yet described in published literature, software must be made available to editors and reviewers. We strongly encourage code deposition in a community repository (e.g. GitHub). See the Nature Portfolio guidelines for submitting code & software for further information.

Data

Policy information about availability of data

All manuscripts must include a data availability statement. This statement should provide the following information, where applicable:

- Accession codes, unique identifiers, or web links for publicly available datasets
- A description of any restrictions on data availability
- For clinical datasets or third party data, please ensure that the statement adheres to our policy

Source data are provided with this paper.
The RNA-seq data generated in this study have been deposited in the GEO database under accession code GSE250125 [https://www.ncbi.nlm.nih.gov/geo/query/acc.cgi?acc=GSE250125]. The proteomics data generated in this study have been deposited in the ProteomeXchange database under accession code PXD042404 [http://proteomecentral.proteomexchange.org/cgi/GetDataset?ID= PXD042404]. OmicsExpressionProteinCodingGenesTPMLogp1.csv was downloaded on 4-5-2023 as part of the DepMap Public 22Q4 data set.

Research involving human participants, their data, or biological material

Policy information about studies with human participants or human data. See also policy information about sex, gender (identity/presentation), and sexual orientation and race, ethnicity and racism.

Reporting on sex and gender	*Use the terms sex (biological attribute) and gender (shaped by social and cultural circumstances) carefully in order to avoid confusing both terms. Indicate if findings apply to only one sex or gender; describe whether sex and gender were considered in study design; whether sex and/or gender was determined based on self-reporting or assigned and methods used. Provide in the source data disaggregated sex and gender data, where this information has been collected, and if consent has been obtained for sharing of individual-level data; provide overall numbers in this Reporting Summary. Please state if this information has not been collected. Report sex- and gender-based analyses where performed, justify reasons for lack of sex- and gender-based analysis.*
Reporting on race, ethnicity, or other socially relevant groupings	*Please specify the socially constructed or socially relevant categorization variable(s) used in your manuscript and explain why they were used. Please note that such variables should not be used as proxies for other socially constructed/relevant variables (for example, race or ethnicity should not be used as a proxy for socioeconomic status). Provide clear definitions of the relevant terms used, how they were provided (by the participants/respondents, the researchers, or third parties), and the method(s) used to classify people into the different categories (e.g. self-report, census or administrative data, social media data, etc.) Please provide details about how you controlled for confounding variables in your analyses.*
Population characteristics	*Describe the covariate-relevant population characteristics of the human research participants (e.g. age, genotypic information, past and current diagnosis and treatment categories). If you filled out the behavioural & social sciences study design questions and have nothing to add here, write "See above."*

Recruitment	*Describe how participants were recruited. Outline any potential self-selection bias or other biases that may be present and how these are likely to impact results.*
Ethics oversight	*Identify the organization(s) that approved the study protocol.*

Note that full information on the approval of the study protocol must also be provided in the manuscript.

Field-specific reporting

Please select the one below that is the best fit for your research. If you are not sure, read the appropriate sections before making your selection.

☒ Life sciences ☐ Behavioural & social sciences ☐ Ecological, evolutionary & environmental sciences

For a reference copy of the document with all sections, see nature.com/documents/nr-reporting-summary-flat.pdf

Life sciences study design

All studies must disclose on these points even when the disclosure is negative.

Sample size	Xenograft tumor assays were performed with at least 5 mice per cohort. The in vivo experiments contained 5 mice for each treatment group and the sample size was chosen to reflect a difference in means of 20% with a power of 90%. In vitro studies were performed in at least duplicate biological samples and across at least triplicate technical replicates. Biological replicates for each experiment were conducted independently at least twice. Each biological replicate was assayed in at least three technical replicates. These sample sizes were chosen to ensure reproducibility within and between experiments.
Data exclusions	No data has been excluded from the analyses.
Replication	All cell viability assays were repeated in triplicate. All immunoblots assay have been repeated in at least duplicate. All microscopy images have been assessed with at least 20 images per condition. Yes, all replicate experiments were successful.
Randomization	As the experimental groups were equally distributed and hence did not require randomization.
Blinding	Blinding was not performed as every experiment has been validated by multiple approaches to corroborate the research findings.

Reporting for specific materials, systems and methods

We require information from authors about some types of materials, experimental systems and methods used in many studies. Here, indicate whether each material, system or method listed is relevant to your study. If you are not sure if a list item applies to your research, read the appropriate section before selecting a response.

Materials & experimental systems

n/a	Involved in the study
☐	☒ Antibodies
☐	☒ Eukaryotic cell lines
☒	☐ Palaeontology and archaeology
☐	☒ Animals and other organisms
☒	☐ Clinical data
☒	☐ Dual use research of concern
☒	☐ Plants

Methods

n/a	Involved in the study
☒	☐ ChIP-seq
☒	☐ Flow cytometry
☒	☐ MRI-based neuroimaging

Antibodies

Antibodies used	ß-Tuband ß-Tubulin (Sigma, T5201; diluted 1:5,000), PTEN (Cell Signaling, 9552; 1:1000), RPS16 (Abcam, ab26159; 1:2500), AP2S1(Abcam, ab128950; 1:250), AP2M1(Abcam, ab75995; 1:250), GapDH(Santa Cruz, sc-365062; 1:5000), ITGB1 (Cell Signaling, 9699; 1:1000), FAK (Cell Signaling, 3285; 1:1000), pFAK (Cell Signaling, 8556; 1:1000), FTH1 (Cell Signaling, 4393; 1:500), TFRC (Cell Signaling, 13113; 1:500), IREB2 (Cell Signaling, 37135s; 1:500), ITGA2 (Thermo Fisher Scientific, MA5-32306; 1:500), ITGA3 (Thermo Fisher Scientific, MA5-28565; 1:250), or ITGB6 (Cell Signaling, 95153; 1:1000).
Validation	PTEN, AP2M1, and AP2S1 antibodies were validated by target gene Knockout experiments in figure 2. All others had been previously published.

Eukaryotic cell lines

Policy information about cell lines and Sex and Gender in Research

Cell line source(s)	All cell lines were purchased Duke Cell culture Facility
Authentication	Cell lines from Duke Cell culture Facility were validated by STR
Mycoplasma contamination	Mycoplasma contamination is tested for monthly in the lab by colorometric assay and all cell lines used in this manuscript were determined negative
Commonly misidentified lines (See ICLAC register)	none

Animals and other research organisms

Policy information about studies involving animals; ARRIVE guidelines recommended for reporting animal research, and Sex and Gender in Research

Laboratory animals	Charles River SCID beige mice Strain 250 (CB17.Cg-PrkdcscidLystbg-J/Crl, Charles River) male and female mice were used for xenograft tumor assays. The animals were housed in a facility at room temperature with 12 hour dark and light cycle.
Wild animals	none
Reporting on sex	Sex was considered in in vivo CRISPR screens. Both male and female mice were used but no gender based effect was detected.
Field-collected samples	none
Ethics oversight	All animal study experimentally have adhered to the Duke Institutional Animal Care and Use Committee protocol and policy.

Note that full information on the approval of the study protocol must also be provided in the manuscript.

Plants

Seed stocks	*Report on the source of all seed stocks or other plant material used. If applicable, state the seed stock centre and catalogue number. If plant specimens were collected from the field, describe the collection location, date and sampling procedures.*
Novel plant genotypes	*Describe the methods by which all novel plant genotypes were produced. This includes those generated by transgenic approaches, gene editing, chemical/radiation-based mutagenesis and hybridization. For transgenic lines, describe the transformation method, the number of independent lines analyzed and the generation upon which experiments were performed. For gene-edited lines, describe the editor used, the endogenous sequence targeted for editing, the targeting guide RNA sequence (if applicable) and how the editor was applied.*
Authentication	*Describe any authentication procedures for each seed stock used or novel genotype generated. Describe any experiments used to assess the effect of a mutation and, where applicable, how potential secondary effects (e.g. second site T-DNA insertions, mosiacism, off-target gene editing) were examined.*
